# Double system contact: The experiences of children-looked-after in the youth justice system

**DOI:** 10.23889/ijpds.v8i2.2329

**Published:** 2023-09-14

**Authors:** Helen Hodges

**Affiliations:** 1 CASCADE / Cardiff University, Cardiff, United Kingdom

## Objectives

To understand the needs and vulnerabilities of this cohort, and identify where there are opportunities for timely and appropriate support from a range of statutory and non-statutory organisations as they navigate the youth justice system.

## Methods

This feasibility study linked external youth justice data from two Welsh local authorities with education, health, social services and family court data held in SAIL Databank. Once combined, it was possible to gain further insights into the nature and extent of contact with other state agencies, and to test for statistically significant differences in the profile of this cohort relative to their peers; in index risk scores across 12 domains and in the trajectory of the probability of further offending behaviour. The latter was considered via a series of hierarchical binary logistical regression models conducted under a Bayesian framework.

## Results

Those with double system contact typically had higher index risk scores than their peers, with the greatest differences being in relation to the Family and Personal Relationships, Living Arrangements, and Emotional and Mental Health domains. In terms of vulnerabilities, the higher rates of additional learning needs were striking, with this potentially contributing to higher levels of non-compliance and more serious consequences. Notably rates of further offending are found to be higher amongst those who are care experienced and/or have additional learning needs, with care experienced children being twice as likely to commit further offences than their peers; more than 2.5 times more likely to breach; twice as likely to return to court and were more than 5 times more likely to spend time in custody.

## Conclusion

Findings, reinforce the need for a more tailored, child first approach in youth justice with other agencies also needing to recognise the impact of prior trauma and adapt their practice in order to appropriately respond to the additional vulnerabilities amongst this cohort.

